# Oleoylethanolamide decreases frustration stress-induced binge-like eating in female rats: a novel potential treatment for binge eating disorder

**DOI:** 10.1038/s41386-020-0686-z

**Published:** 2020-04-30

**Authors:** Adele Romano, Maria Vittoria Micioni Di Bonaventura, Cristina Anna Gallelli, Justyna Barbara Koczwara, Dorien Smeets, Maria Elena Giusepponi, Marialuisa De Ceglia, Marzia Friuli, Emanuela Micioni Di Bonaventura, Caterina Scuderi, Annabella Vitalone, Antonella Tramutola, Fabio Altieri, Thomas A. Lutz, Anna Maria Giudetti, Tommaso Cassano, Carlo Cifani, Silvana Gaetani

**Affiliations:** 1 Department of Physiology and Pharmacology “V. Erspamer”, Sapienza University of Rome, P.le Aldo Moro 5, 00185 Rome, Italy; 2 School of Pharmacy, Pharmacology Unit, University of Camerino, via Madonna delle Carceri, 9, 62032 Camerino, MC Italy; 3 Department of Biochemical Sciences “A. Rossi Fanelli”, Sapienza University of Rome, P.le Aldo Moro 5, 00185 Rome, Italy; 4 Institute of Veterinary Physiology Vetsuisse Faculty, University of Zurich, Winterthurerstrasse 260, 8057 Zurich, Switzerland; 5 Department of Biological and Environmental Sciences and Technologies, University of Salento, Lecce, Italy; 6 Department of Clinical and Experimental Medicine, University of Foggia, Via L. Pinto 1, 71122 Foggia, Italy

**Keywords:** Feeding behaviour, Pharmacology

## Abstract

Binge eating disorder (BED) is the most frequent eating disorder, for which current pharmacotherapies show poor response rates and safety concerns, thus highlighting the need for novel treatment options. The lipid-derived messenger oleoylethanolamide (OEA) acts as a satiety signal inhibiting food intake through the involvement of central noradrenergic and oxytocinergic neurons. We investigated the anti-binge effects of OEA in a rat model of binge-like eating, in which, after cycles of intermittent food restrictions/refeeding and palatable food consumptions, female rats show a binge-like intake of palatable food, following a 15-min exposure to their sight and smell (“frustration stress”). Systemically administered OEA dose-dependently (2.5, 5, and 10 mg kg^−1^) prevented binge-like eating. This behavioral effect was associated with a decreased activation (measured by mapping the expression of *c-fos*, an early gene widely used as a marker of cellular activation) of brain areas responding to stress (such as the nucleus accumbens and amygdala) and to a stimulation of areas involved in the control of food intake, such as the VTA and the PVN. These effects were paralleled, also, to the modulation of monoamine transmission in key brain areas involved in both homeostatic and hedonic control of eating. In particular, a decreased dopaminergic response to stress was observed by measuring dopamine extracellular concentrations in microdialysates from the nucleus accumbens shell, whereas an increased serotonergic and noradrenergic tone was detected in tissue homogenates of selected brain areas. Finally, a decrease in corticotropin-releasing factor (CRF) mRNA levels was induced by OEA in the central amygdala, while an increase in oxytocin mRNA levels was induced in the PVN. The restoration of a normal oxytocin receptor density in the striatum paralleled the oxytocinergic stimulation produced by OEA. In conclusion, we provide evidence suggesting that OEA might represent a novel potential pharmacological target for the treatment of binge-like eating behavior.

## Introduction

Binge eating disorder (BED) is the most frequent eating disorder occurring in 2–5% of the adult population, with a higher prevalence among women than men [1–3]. BED is characterized by uncontrollable and compulsive episodes of excessive consumption of highly palatable food (HPF) accompanied by a strong sense of loss of control, feeling of shame, guilt, disgust, and anxiety. The combination of dieting and stress is a common trigger for BED [4, 5], which shares a variety of commonalities with drug addiction [6]. A large body of evidence suggests that the neurobiological mechanisms of BED converge on the activation of the mesocorticolimbic dopamine (DA) system [7, 8], as well as on brain serotonin (5-HT) and noradrenaline (NA) signaling [9–12]. Lisdexamfetamine, a prodrug of d-amphetamine, is the first medication approved for BED treatment in the United States that acts primarily by enhancing brain dopaminergic and noradrenergic neurotransmission [13]. Its most common side effects include insomnia, weight loss, and headache [14], and its highest limit derives from serious adverse effects in patients suffering from cardiomyopathies, which are quite frequent comorbidities [15, 16]. Furthermore, being a psychostimulant, there is a considerable risk of abuse. Other treatments tested for BED lack sufficient efficacy, and are complicated by high relapse rates and a wide range of side effects [17, 18].

**Fig. 1:**
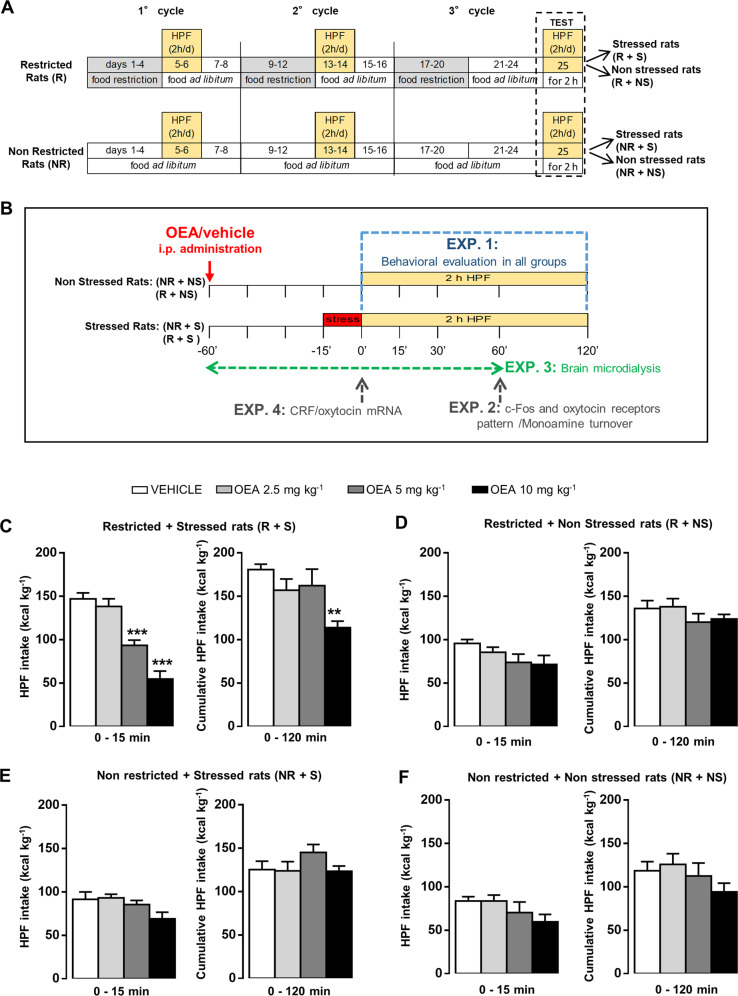
OEA treatment selectively prevented binge-like eating in a dose-dependent manner. **a** Female rats were exposed (restricted rats, R) or not exposed (non restricted, NR) to three 8-day cycles of intermittent food restriction (66% of chow intake), occurring on days 1–4, and free-feeding condition allowed on days 5–8 of each cycle. During the *ad libitum* condition of days 5–6 and 13–14 of the first two cycles, both NR and R rats were given access to HPF for 2 h during the light phase. On day 25, both R and NR rats were exposed (R + S and NR + S) or not exposed (R + NS and NR + NS) to frustration stress. **b** EXP. 1: on test day (day 25), after the third cycle, both NR and R rats were administered with vehicle (veh) or three different doses of OEA (2.5, 5, and 10 mg kg^−1^, intraperitoneal injection (i.p.)). Forty-five minutes after treatments, both NR and R rats were exposed (stressed: NR + S and R + S) or not exposed (non stressed: NR + NS and R + NS) to a 15-min stress procedure. One hour after the respective treatments, rats were given free access to HPF for 120 min, and food intake was monitored. EXP. 2: NR + S and R + S rats were administered with veh or OEA (10 mg kg^−1^ i.p.), and were allowed to consume the HPF only for 60 min. At the end of this procedure, rats were sacrificed, their brains immediately collected for immunohistochemical evaluation of the pattern of c-Fos expression, oxytocin receptor expression, and HPLC analyses of monoamine turnover. EXP. 3: NR + S and R + S rats were administered with veh or OEA (10 mg kg^−1^, i.p.), and underwent brain microdialysis in the AcbSh for the analysis of DA extracellular levels (the detailed paradigm of the microdialysis experiment is described in the legend of Fig. 3). EXP. 4: NR + S and R + S rats were administered with veh or OEA (10 mg kg^−1^, i.p.), and immediately sacrificed at the end of the stress procedure. Their brains were collected for in situ hybridization analysis of CRF and oxytocin mRNA. HPF intake (kcal kg^−1^) during the first 15 min (left) and the total 120 min (right) test session after vehicle (veh) or three different doses of OEA (2.5, 5, and 10 mg kg^−1^ i.p.) administration to R + S (**c**, restricted + stressed, *N* = 31), R + NS (**d**, restricted + non stressed, *N* = 28), NR + S (**e**, non restricted + stressed, *N* = 26), and NR + NS (**f**, non restricted + non stressed, *N* = 30). Data are expressed as mean ± SEM. **P < 0.01; ***P < 0.001 vs R + S veh (Bonferroni’s test for multiple comparisons).

Several observations have been accumulated, suggesting that the lipid-derived messenger oleoylethanolamide (OEA) might represent a better pharmacological target for the treatment of BED [19–21]. OEA reduces food intake and body weight gain in obese rodents and humans [20, 22], mainly through the activation of peroxisome proliferator-activated receptor-alpha [23], with a mechanism that appears behaviorally selective [24, 25] and associated with the activation of key brain areas, including the nucleus of the solitary tract, the area postrema [26, 27], the tuberomammillar [28], and paraventricular (PVN) [29] nuclei, where noradrenergic [30], histaminergic [31], and oxytocinergic [32] neurons play a necessary role. Interestingly, it has been demonstrated that OEA treatment is able to restore a physiologic sensitivity to the rewarding properties of fat in diet-induced obese mice [33], and it is able to exert anti-depressant-like effect in different animal laboratory models [34, 35], by regulating the level of both 5-HT and NA in the brain [36]. In this study, we used a rat model of binge-like palatable food consumption [37–41] to test the hypothesis that OEA might be a novel target for BED treatment. In this model, young female rats are subjected to three 8-day cycles (total 24 days) of intermittent food restriction/refeeding (Fig. 1a). On the day of the experiment (day 25), these rats show binge-like HPF consumption after the exposure to a 15-min “frustration stress”, consisting of the sight and smell of HPF placed out of reach [42–46] (Supplementary Fig. S1B). We will refer in the text to dietary-restricted (R) vs not-restricted (NR) rats and exposed-to-stress (S) vs not-exposed-to stress (NS) rats (Fig. 1a). In this model, we investigated the anti-binging acute effects of OEA (2.5, 5, or 10 mg kg^−1^, i.p.) on HPF intake, and analyzed the neurobiological bases of these effects by focusing on different endpoints. These include the brain pattern of c-Fos expression, DA extracellular release in the shell of the nucleus accumbens (AcbSh), monoamine tissue concentrations/turnovers in selected brain regions, corticotropin-releasing factor (CRF), and oxytocin mRNA levels in the central amygdala (CeA) and PVN and, finally, oxytocin receptor immunoreactivity in selected brain areas (Fig. 1b). In all these neurochemical analyses, we focused our attention on the stressed groups (R + S vs NR + S), comparing the effects of OEA vs vehicle treatment. The rationale of this choice is based on the observation that intermittent caloric restriction is the predisposing condition that allows stress to act as a trigger (R + S), whereas the *ad libitum* feeding condition represents the baseline control, in which stress is ineffective (NR + S), thus also providing the control for the stress effect (Supplementary Fig. S1B).

## Materials and methods

### Binge eating model

One-hundred and eighty-four female Sprague Dawley rats (Charles River, Italy), 200–225 g at the beginning of the experiments, were housed under a 12-h light/dark cycle (lights on at 8:00 a.m.), at constant temperature (20–22° C) and humidity (45–55%), and with access to food and water ad libitum for 2 weeks before the experiments. According to the dietary schedule, the rats were given standard food pellets (4RF18, Mucedola, 2.6 kcal/g) or HPF (3.63 kcal/g) consisting of a paste prepared by mixing Nutella (Ferrero^®^) chocolate cream (5.33 kcal/g; 56, 31, and 7% from carbohydrate, fat, and protein, respectively), grounded food pellets (4RF18), and water in the following w/w/w percent ratio: 52% Nutella, 33% food pellets, and 15% water.

The procedure for binge eating induction was performed according to our previous studies [37, 47, 48]. Briefly, two groups of female rats were housed individually in metal cages (30 × 30 × 30 cm) and exposed (or not exposed) for 24 days to three 8-day cycles of intermittent food restriction (66% of chow intake on days 1–4 and free feeding on days 5–8 of each cycle), during which they were given access to HPF for 2 h during the light cycle between 10:00 a.m. and 12:00 a.m. (2 h after the onset of the light cycle) on days 5–6 and 13–14 of the first two cycles (total of four exposures). Although this intermittent caloric restriction caused body weight fluctuations during the three cycles, on the test day, similar body weights (S2 Supplementary results and Supplementary Fig. S1A) were detected in all rats.

On the test day (day 25), at 10 a.m., half of the rats in each group were subjected to a 15-min frustration stress, consisting of the exposure to HPF placed out of reach. During this 15-min period, the rats could smell and see the HPF and repeatedly attempted to reach it. The second half of rats in each group were not exposed to the stress manipulation. Therefore, we will refer in this paper to dietary restricted (R) vs non restricted (NR) rats and exposed to stress (S) vs non exposed to stress (NS) rats. After 15 min of stress exposure, the HPF was placed inside the cage for all rats. In accordance with our previous studies, binge eating behavior occurred in R + S rats (Supplementary Fig. S1B), as demonstrated by the immediate and persistent consumption of a larger amount of HPF within the first 15-min access, with respect to the other groups (S2 Supplementary results). Vaginal smears were collected at the end of the experiments to exclude from the results rats in the estrous phase, since we previously observed that binge eating does not occur during the estrous phase of female rats [49, 50].

The experimental procedure is depicted in Fig. 1a. This paradigm was used in four different experiments, in which the consumption of the HPF was allowed for 120 min, 60 min, or 0 min, depending on the endpoints analyzed (Fig. 1b). All experiments were carried out in accordance with the European directive 2010/63/UE governing animal welfare, and with the Italian Ministry of Health guidelines for the care and use of laboratory animals.

### Experiment 1: effect of OEA on stress-induced binge eating

The first set of rats (*N* = 144) was divided into 16 groups (*N* = 9 per group) in a 2 (history of intermittent food restriction: yes (R), no (NR) rats) × 2 (stress during testing: yes (S), no (NS) rats) × 4 (OEA dose: 0, 2.5, 5, and 10 mg kg^−1^) factorial design, to evaluate the behavioral effects of OEA during the test day. To this aim, OEA or vehicle were administered 1 h before the access to HPF; rats were exposed (or not exposed) to the 15-min frustration stress, and once they had access to the HPF, the intake was measured at the following time points (15, 30, 60, and 120 min). The experimental paradigm is depicted in Fig. 1b (EXP. 1). After testing, 29 rats were excluded from statistical analyses because they were in the estrous phase.

### Experiment 2: effects of OEA on the pattern of c-Fos, oxytocin receptor expression, and on monoamine turnover

Previous studies demonstrated that the effect of OEA on food intake is paralleled by a selective induction of *c-fos*, an immediate early gene widely used as a marker of cellular activation, at the level of the hypothalamus (HYPO) and brainstem [26, 27, 29], key regions involved in the control of feeding [51]. Here, we have expanded those findings by examining the impact of OEA (10 mg kg^−1^) on the brain pattern of c-Fos immunostaining in response to 60 min of HPF consumption in female rats with different diet histories and exposed to acute stress (R + S vs NR + S, Fig. 1b, EXP. 2). In this experiment, we tested the effects of the highest dose (OEA 10 mg kg^−1^ i.p.), based on the observations made in EXP. 1.

Moreover, we evaluated whether the interaction between food restriction and stress exposure is accompanied by alteration of oxytocin receptor immunoreactivity in selected brain regions, and whether OEA treatment is able to affect this endpoint.

As a further aim of this experiment (Fig. 1b, EXP. 2), we analyzed the effects of OEA on tissue concentrations of monoamines (DA, 5-HT, and NA) and their main metabolites in the principal neural nodes that control different aspects of food intake in the brain.

The immunohistochemistry experiment and monoamine analyses were performed according to our previous studies [27, 52]. The detailed protocols are described in the sections S1.1 of Supplementary Materials and Methods.

### Experiment 3: effects of OEA on DA transmission in the AcbSh

To investigate whether OEA would decrease the central dopaminergic response to appetitive/reinforcing stimuli, we performed in vivo microdialysis experiment to evaluate DA extracellular concentration at the level of the AcbSh in R + S and NR + S rats (Fig. 1b, EXP. 3), according to the protocol used in our previous study [53, 54]. To this aim, a new set of rats (*N* = 40) was divided into R and NR groups, according to the protocol described for EXP. 1, and underwent the procedure for microdialysis experiment. The detailed protocol is described in the section S1.2 of Supplementary Materials and Methods and in the legend of Fig. 3.

### Experiment 4: effects of OEA on CRF and oxytocin mRNA

In situ hybridization was performed in brain slices obtained from R + S and NR + S rats according to the protocol reported in our previous studies [26, 29, 55]. The detailed protocol is described in the section S1.3 of Supplementary Materials and Methods.

### Statistical analyses

Statistical analysis is described in the section S1.4 of Supplementary Information.

## Results

### OEA treatment selectively prevented binge-like eating in a dose-dependent manner

We found that acute treatment with OEA, systemically administered to rats 1 h before giving access to HPF (Fig. 1b, EXP. 1), selectively prevented binge-like eating of R + S rats (Fig. 1c), without altering feeding behavior in the other experimental groups (Fig. 1d–f). In particular, OEA decreased frustration stress-induced HPF overconsumption in a dose- and time-dependent manner, with the strongest and long-lasting effect observed at the dosage of 10 mg kg^−1^ i.p. (Fig. 1c). The intermediate dose of OEA (5 mg kg^−1^ i.p.) was effective only at the 15-min time point, while the lowest dose of OEA was ineffective. The results obtained from ANOVA showed a significant effect of treatment in the session time 0–15 min (*F*_treatment_ = 29.763, df = 3/27, *P* < 0.001) and in 0–120 min (*F*_treatment_ = 5.758, df = 3/27, *P* < 0.01). Significant differences among groups evaluated by the post hoc analyses are indicated in Fig. 1c.

### OEA treatment affected the brain pattern of c-Fos expression in bingeing rats

**Fig. 2:**
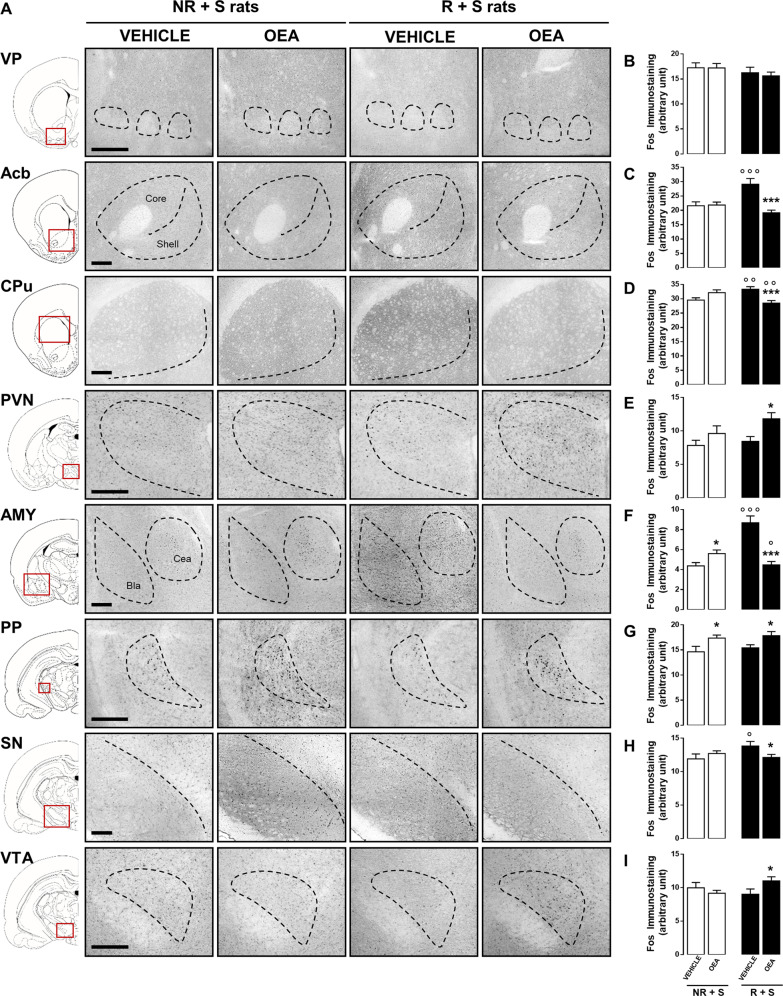
OEA treatment affected the brain pattern of c-Fos expression in bingeing rats. Representative photomicrographs (scale bar = 500 μm, **a**) showing c-Fos immunostaining within the ventral pallidum (VP), nucleus accumbens (Acb), caudate putamen (CPu), paraventricular nucleus (PVN), amygdala (AMY), pedunculopontine nucleus (PP), substantia nigra (SN), and ventral tegmental area (VTA) in brain slices collected from both NR + S (non restricted + stressed) and R + S (restricted + stressed) rats treated with either vehicle (veh) or OEA (10 mg kg^−1^, i.p.) and sacrificed 120 min after treatment. Semiquantitative densitometric analysis of c-Fos expression within the VP (**b**), Acb (**c**), CPu (**d**), PVN (**e**), AMY (**f**), PP (**g**), SN (**h**), and VTA (**i**) of NR + S and R + S rats treated with either veh or OEA (10 mg kg^−1^, i.p.) and sacrificed 120 min after treatment. Data are expressed as mean ± SEM. *P < 0.05; ***P < 0.001 vs veh in the same diet regimen group; °*P* < 0.05; °°*P* < 0.01; °°°*P* < 0.001 vs NR + S in the same treatment group (Tukey’s post hoc test, *N* = 3).

The semiquantitative analyses of immunostaining optical densities revealed that the interaction between intermittent food restriction and stress exposure induced an increase of c-Fos expression in the nucleus accumbens (Acb), caudate putamen (CPu), amygdala (AMY), and substantia nigra (SN) of bingeing rats (R + S veh), with respect to non-bingeing rats (NR + S veh), and that OEA treatment completely prevented such increase (Fig. 2c, d, f, h). Conversely, c-Fos expression within the PVN, pedunculopontine nucleus (PP), and ventral tegmental area (VTA) (Fig. 2e, g, i) was unchanged in bingeing rats (R + S veh), with respect to non-binging rats (NR + S veh), but significantly increased by OEA treatment (R + S-OEA vs R + S veh), which induced a similar effect also in the AMY and PP of NR + S rats (Fig. 2f, g, respectively). No difference was observed within the ventral pallidum nucleus among all rat groups (Fig. 2b). The results from the two-way ANOVA analyses of c-Fos expression are reported in Table S1; the results obtained from the post hoc analyses are reported in Fig. 2.

### OEA treatment affected monoaminergic system in bingeing rats

The results obtained from two-way ANOVA analyses are reported in Table S2, while the results from the post hoc analyses (Tukey’s test) are reported in Table 1. Overall, the results (Table 1) revealed that OEA treatment affected mainly monoaminergic tissue concentration/turnover in bingeing rats, rather than in NR + S rats. In fact, in NR + S rats, the effects of OEA treatment included only an increase in NA and DA concentration within the HYPO and VTA, respectively, and an increase of 5-HT turnover in the Acb. Analyzing the results obtained from vehicle- administered rats, bingeing rats (R + S veh) showed an increased DA turnover in the medial prefrontal cortex (mPFC) and AMY, as well as increased 5-HT turnover and 5-HT tissue concentration in the AMY and HYPO, respectively, as compared with non-bingeing rats (NR + S veh). The increased turnovers observed in the mPFC and AMY of bingeing rats resulted in complete prevention by OEA treatment, which increased DA and 5-HT concentrations in the mPFC. The latter effect was accompanied by a decrease of 5-HT turnover in the mPFC of R + S OEA rats, with respect to their vehicle-treated controls. DA tissue concentrations were affected by OEA treatment also in Acb (where it decreased) and VTA (where it increased) of R + S rats, without producing any effect on DA turnover. Similarly, OEA administration to R + S rats caused a marked increase of 5-HT tissue concentrations in Acb, hippocampus (HIPP), VTA, and locus ceruleus (LC), without affecting 5-HT turnover in these areas. Finally, OEA treatment caused a significant increase of NA concentration in the CPu, HYPO, VTA, and LC.

**Table 1:**
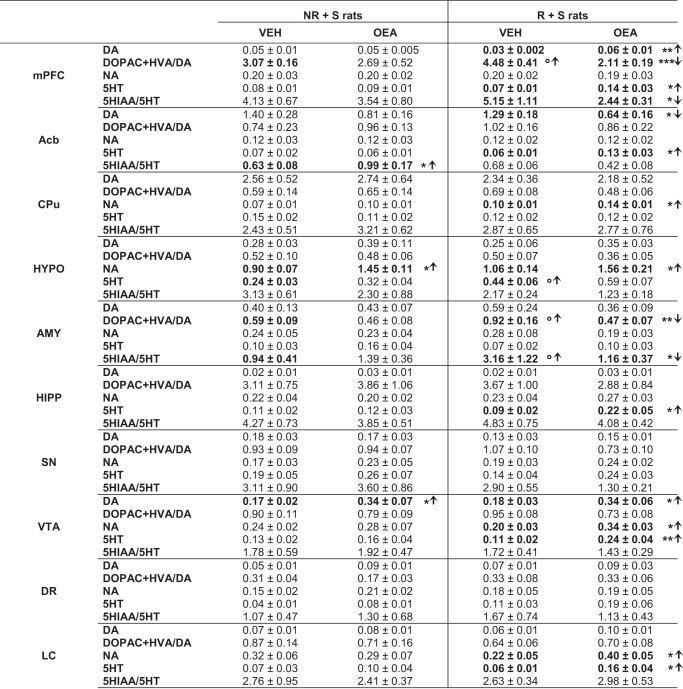
Tissue monoamine concentrations (ng mg^–1^ of wet tissue) and monoamine turnover in selected brain areas.

### OEA treatment dampened AcbSh DA release induced by stress exposure or amphetamine challenge

**Fig. 3:**
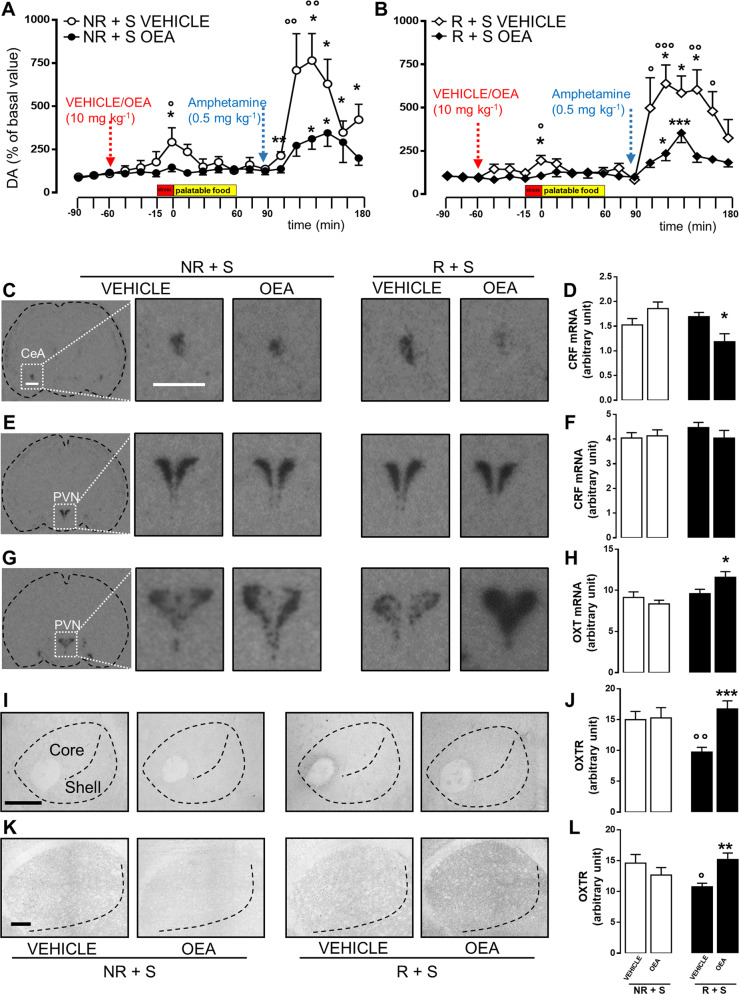
OEA treatment dampened AcbSh DA release induced by stress exposure or amphetamine challenge. OEA treatment affected CRF, oxytocin mRNA levels, and oxytocin receptor expression in bingeing rats. Time course of extracellular DA levels (expressed as % of basal values) measured in the nucleus accumbens shell of NR + S (non restricted + stressed, **a**, *N* = 9–11) and R + S (restricted + stressed, **b**, *N* = 6–9) rats during microdialysis experiment. The first three samples were collected before treating rats with vehicle (veh) or OEA (10 mg kg^−1^, i.p.) and used as baseline (NR + S baseline = 225.5 ± 43.66; R + S baseline = 205.1 ± 21.07, no statistically significant difference); 45 min after treatment, rats were subjected to the stress procedure for 15 min and subsequently received the HPF for 60 min. Thirty minutes after the end of HPF exposure, rats were administered with amphetamine (0.5 mg kg^−1^, subcutaneous injection (s.c.)). Data are expressed as mean ± SEM. *P < 0.05; **P < 0.01; ***P < 0.001 vs the mean of the first three samples (basal values) within the same group (Dunnett’s multiple-comparison test). °*P* < 0.05; °°*P* < 0.01; °°°*P* < 0.001 vs OEA-treated rats in the same time point of the same diet regimen group (Bonferroni’s test for between-group comparisons). Red arrow: veh or OEA (10 mg kg^−1^, i.p.) administration; blue arrow: amphetamine administration (0.5 mg kg^−1^, s.c.). Representative in situ hybridization images (scale bar = 1 mm) of CRF mRNA expression within the central amygdala (CeA, **c**), CRF, and oxytocin mRNA in the paraventricular nucleus (PVN, **e**, **g**) of NR + S (non restricted + stressed) and R + S (restricted + stressed) rats treated with either vehicle (veh) or OEA (10 mg kg^−1^, i.p.), and sacrificed 60 min after the treatment. Semiquantitative densitometric analyses of CRF mRNA in the CeA (**d**), and CRF and oxytocin mRNA in the PVN (**f**, **h**, respectively) of NR + S and R + S rats treated with either veh or OEA (10 mg kg^−1^, i.p.), and sacrificed 60 min after treatment. Data are expressed as mean ± SEM. *P < 0.05 vs veh in the same diet regimen group (Tukey’s post hoc test, *N* = 4–6). Representative photomicrographs (scale bar = 500 μm) showing oxytocin receptor (OXTR) immunostaining within the nucleus accumbens (core and shell, **i**) and the caudate putamen (**k**) in brain slices collected from both NR + S (non restricted + stressed) and R + S (restricted + stressed) rats treated with either vehicle (veh) or OEA (10 mg kg^−1^, i.p.) and sacrificed 120 min after treatment. Semiquantitative densitometric analysis of oxytocin receptor expression within the nucleus accumbens (**j**) and caudate putamen (**l**) of NR + S and R + S rats treated with either veh or OEA (10 mg kg^−1^, i.p.) and sacrificed 120 min after treatment. Data are expressed as mean ± SEM. **P < 0.01; ***P < 0.001 vs veh in the same diet regimen group; °*P* < 0.05; °°*P* < 0.01 vs NR + S in the same treatment group (Tukey’s post hoc test, *N* = 3).

In agreement with previous reports [56–59], in both rat groups treated with vehicle, dialysate DA levels significantly exceeded the basal values in response to stress exposure or to amphetamine challenge, with no change induced by HPF consumption (Fig. 3a, b). The first increase in response to stress exposure was transitory (15 min) and reached 292 and 194% in NR + S veh and R + S vehicle rats, respectively; the second increase was long-lasting (about 90 min) and reached maximum values of 764% and 638%, in non-bingeing and bingeing vehicle-treated rats, respectively. OEA administration did not alter DA basal levels in either experimental groups, but significantly attenuated the increase in DA efflux evoked by frustration stress and by amphetamine challenge, independently from the history of caloric restriction (Fig. 3a, b).

The results obtained by the two-way ANOVA for repeated measures revealed a significant effect of time, treatment, and a significant interaction between the two factors (R + S: *F*_time_ = 17.252, df = 18/234, *P* < 0.001, *F*_treatment_ = 27.407, df = 1/13, *P* < 0.001, and *F*_interaction_ = 5.018, df = 18/234, *P* < 0.01; NR + S: *F*_time_ = 15.216, df = 18/324, *P* < 0.001, *F*_treatment_ = 6.154, df = 1/18, *P* < 0.05, and *F*_interaction_ = 3.142, df = 18/324, *P* < 0.05). The results obtained by post hoc tests are reported in Fig. 3a, b.

### OEA treatment affected CRF and oxytocin mRNA levels in bingeing rats

We previously demonstrated a crucial role of oxytocinergic neurotransmission in mediating the hypophagic effect of OEA [29], as well as the pivotal role played by CRF system in sustaining binge eating behavior in the experimental model used in the present study [47]. Since both oxytocin and CRF can be affected by stress and food intake, we assessed the “pure” effects of OEA on stress response without the potential impact of caloric consumption, to evaluate whether the anti-bingeing effects of OEA might be attributed to a reduced effect of stress exposure. To this aim, we measured both CRF and oxytocin mRNA levels by in situ hybridization in the brains of NR + S and R + S rats treated with either OEA or vehicle and sacrificed at the end of the stress exposure (Fig. 1b, EXP. 4).

As shown in the representative autoradiography reported in Fig. 3c, e, CRF mRNA signal was detected and measured in the CeA and the PVN. The results of the densitometric analyses of CeA were statistically analyzed by two-way ANOVA that revealed no effect of caloric restriction and no effect of treatment, but a significant interaction between the two factors (*F*_interaction_ = 9.491, df = 1/19, *P* < 0.01). Post hoc analyses demonstrated that OEA treatment reduced CRF mRNA in the CeA of bingeing rats (Fig. 3d), whereas the two-way ANOVA in the PVN revealed no significant effect (Fig. 3f). As shown in the representative autoradiography reported in Fig. 3g oxytocin mRNA signal was detected and measured in the PVN. Two-way ANOVA analyses revealed a significant effect of food restriction (*F*_restriction_ = 9.897, df = 1/20, *P* < 0.01), no effect of treatment, and a significant interaction between the two factors (*F*_interaction_ = 5.544, df = 1/20, *P* < 0.05). The post hoc analyses demonstrated that oxytocin mRNA expression was significantly increased in bingeing rats treated with OEA (Fig. 3h).

### OEA treatment affected oxytocin receptor expression in selected brain areas of bingeing rats

Oxytocin receptors are abundantly expressed in the striatum [60], where they control, through different mechanisms, dopaminergic neurotransmission. Therefore, as the last step of our study, we investigated whether bingeing rats show different oxytocin receptor immunoreactivity in the CPu and Acb (Fig. 3i, k), as compared with non-bingeing rats, and whether OEA treatment might affect such parameters. The results obtained by the semiquantitative densitometric analyses of optical densities revealed that binge eating behavior in R + S rats was associated with a reduced oxytocin receptor expression within both the dorsal (CPu) and the ventral (Acb) striatum, and that OEA treatment completely restored such decrease, reporting oxytocin receptor immunoreactivity to the level observed in NR + S rats (Fig. 3j, l). In particular, two-way ANOVA analyses of oxytocin receptor expression within the Acb revealed a significant effect of treatment (*F*_treatment_ = 7.445, df = 1/11, *P* < 0.05), no effect of food restriction, and significant interaction between the two factors (*F*_interaction_ = 6.363, df = 1/11, *P* < 0.05). The same effect was observed within the CPu (*F*_interaction_ = 8.479, df = 1/11, *P* < 0.05) (Fig. 3j, l).

## Discussion

### OEA prevents binge-like palatable food consumption

This study demonstrates that OEA prevents binge-like palatable food consumption induced by stress in female rats with a history of food restriction (R + S rats), supporting the hypothesis that this lipid signal might represent a potential target for the development of more efficacious and safer treatments for BED or for other eating disorders characterized by binge episodes. The effect of OEA was dose- and time-dependent, being long-lasting at the dose of 10 mg kg^−1^ i.p. According to previous reports from our laboratory and from other research groups [25–27, 29, 30], this dosage of OEA is able to induce satiety in both free-feeding and food-deprived rats, without causing motor impairment, malaise, pain, or hormonal and body temperature alterations. In the present study, we were unable to detect any significant effect of OEA on feeding behavior in the other three experimental groups, which did not show binge-like palatable food intake within the timeframe of the experiment. This observation suggests that the anti-binge effects of OEA, rather than the expression of satiety induction, might likely be the consequence of the selective inhibition of “hedonic hunger” [61, 62].

### OEA modulates monoaminergic tone in key brain areas

Based on this evidence, to investigate the neurobiological counterparts of OEA effect on binge eating, we focused our attention on the so-called “DA motive system” controlling the reinforcement and motivational aspects of feeding, including compulsive eating [63]. Our choice is based on previous findings demonstrating the capability of OEA to counteract different addiction-related behaviors, by acting within this system [64–66].

The results obtained by analyzing c-Fos expression in brain areas that partake directly or indirectly to this system suggest that the anti-binge effects of OEA are associated with its ability to dampen the “trigger” effects of stress in R + S rats. This action is accomplished by “normalizing” the activity of areas responding to stress exposure (Acb, CPu, SN, and AMY), and by increasing the activation of areas involved in the control of food intake (VTA and PVN). The effect observed in the Acb was associated with a decrease of DA tissue levels induced by OEA administration to R + S rats. Expanding this latter result, data obtained from microdialysis of the AcbSh revealed that OEA dampened DA response to stress and to amphetamine challenge in both R + S and NR + S rats. Previous studies from Tellez and collaborators have demonstrated that OEA treatment restored a normal dopaminergic nigrostriatal response to fat intake in diet-induced obese mice [33]. The results of our experiments expand their observation, demonstrating that OEA is able to restore a normal dopaminergic response not only to food consumption but also to stress-induced appetitive motivation. However, in our study, the attenuation of AcbSh DA release evoked by OEA in response to stress exposure did not perfectly parallel the selective behavioral effects. In fact, although OEA induced the same effect on DA release in the Acb of both R + S and NR + S rats, it significantly inhibited HPF consumption only in the R + S group, thus suggesting the involvement of other possible systems.

Based on previous observations, we hypothesized that these systems might include 5-HT, NA, CRF, and oxytocin. In support of this hypothesis, we found that in R + S rats, OEA selectively enhanced NA levels in the CPu, VTA, and LC, as well as it increased 5-HT tissue levels in most of the brain areas analyzed (mPFC, Acb, HIPP, VTA, and LC). These results are in accordance with previous studies showing that OEA exerts anti-depressant-like effects in different animal models [34], by regulating 5-HT and NA levels [36], and suggest that the anti-binge effects of OEA might occur, at least in part, by promoting a high serotonergic/noradrenergic tone.

### OEA affects central CRF and oxytocinergic systems in bingeing rats

The results obtained within the AMY, where OEA significantly decreased stress-induced c-Fos activation in bingeing rats, prompted us to investigate whether OEA might influence the CRF system, known to coordinate the frustration-stress response in a rat model [48]. In agreement with this notion, and overlapping with the trend of c-Fos induction in the AMY, we found that OEA decreased CRF mRNA level in the CeA of R + S rats, without producing any effect on the PVN, and without affecting the same parameters in NR + S rats. The results confirm previous findings demonstrating that hypothalamic CRF system is not sufficient to account for binge-like HPF consumption in our BED model [47, 48], and that CRF in the CeA plays a key role in other models of excessive palatable food consumption [67–69]. These latter observations were further supported by the findings that treatments with CRF antagonists can prevent binge eating by interacting with CRF receptors in bed nucleus of the stria terminalis [47, 48, 70] and CeA [67–69], rather than at hypothalamic levels.

Furthermore, we hypothesized that the ability of OEA to increase c-Fos expression within the PVN of R + S rats might be linked to the activation of oxytocinergic neurons [30]. In agreement with our previous studies [29, 32], we found that OEA treatment increased oxytocin mRNA levels in the PVN of R + S rats, without producing any effect on NR + S rats. We expanded this notion by analyzing also oxytocin receptor expression. We observed a reduced immunoreactivity for oxytocin receptors within both the CPu and the Acb of R + S rats, as compared with NR + S rats. This result suggests a hypofunctionality of the oxytocinergic system at the level of these two brain regions that might be associated with the compulsive eating in response to stress. In fact, it is well demonstrated that oxytocin transmission has a key role in attenuating stress responses by exerting inhibitory actions on the HPA axis, sympathetic activity, and anxiety-related behavior during exposure to stressful stimuli [71–73]. Our functional hypothesis is that cycles of food restriction might attenuate oxytocin sensitivity in R + S rats; OEA treatment might be able to rescue this alteration by normalizing oxytocin receptor density and stimulating oxytocin release from the PVN, thus overall increasing oxytocin transmission in bingeing rats. Such effect might contribute, in turn, to the reduced CRF synthesis in the CeA, as supported by several findings demonstrating a genomic effect of oxytocin on CRF gene expression [74].

## Conclusions

We provide evidence that OEA exerts a selective inhibitory effect on binge-like eating behavior in female rats, and that such effect is associated with the modulation of several neurochemical endpoints measured within the hedonic–homeostatic circuits that involve monoaminergic systems, as well as two key neuropeptidergic systems, namely CRF and oxytocin. Although further studies should investigate the causative link between these observations, our findings broaden the current knowledge of the role played by OEA in the caloric–homeostatic control system, and support the hypothesis that OEA might represent a novel potential pharmacological target for the treatment of aberrant eating patterns.

## Funding and disclosure

This research was supported by grants (PRIN 2012JTX3KL to CC and SG; PRIN2015KP7T2Y to CC and CS; RBFR12DELS to SG and CC) of the Italian Ministry of Education, University and Research. The authors declare no competing interests.

## Supplementary information

Supplementary information
