# Supplementary material for: Oleoylethanolamide decreases frustration stress-induced binge-like eating in female rats: a novel potential treatment for binge eating disorder
Source: Neuropsychopharmacology. 2020 Apr 30;45(11):1931–41. doi: 10.1038/s41386-020-0686-z (PMC7609309; doi:10.1038/s41386-020-0686-z)
Supplement: Supplementary file 1 — Supplementary information [file 41386_2020_686_MOESM1_ESM.docx]

**Supplementary Information for**

**Oleoylethanolamide decreases frustration stress-induced binge-like eating in female rats: a novel potential treatment for binge eating disorder**

Adele Romano^a*^, Maria Vittoria Micioni Di Bonaventura^b*^, Cristina Anna Gallelli^a^, Justyna Barbara Koczwara^a^, Dorien Smeets^a^, Maria Elena Giusepponi^b^, Marialuisa De Ceglia^a^, Marzia Friuli^a^, Emanuela Micioni Di Bonaventura^b^, Caterina Scuderi^a^, Annabella Vitalone^a^, Antonella Tramutola^c^, Fabio Altieri^c^, Thomas A Lutz^d^, Anna Maria Giudetti^e^, Tommaso Cassano^f^, Carlo Cifani^b#^ and Silvana Gaetani^a#^

^a^Dept. of Physiology and Pharmacology “V. Erspamer”, Sapienza University of Rome, P.le Aldo Moro 5, 00185 Rome Italy; ^b^School of Pharmacy, Pharmacology Unit, University of Camerino, via Madonna delle Carceri, 9, 62032 Camerino, Italy; ^c^Dept. of Biochemical Sciences “A. Rossi Fanelli”, Sapienza University of Rome, P.le Aldo Moro 5, 00185 Rome Italy; ^d^Institute of Veterinary Physiology Vetsuisse Faculty, University of Zurich Winterthurerstrasse 260, 8057 Zurich Switzerland; ^e^Dept. of Biological and Environmental Sciences and Technologies, University of Salento, 73100 Lecce, Italy; ^f^Dept. of Clinical and Experimental Medicine, University of Foggia, Via L. Pinto 1, 71122 Foggia, Italy

**Correspondence to:**

Prof. Carlo Cifani at University of Camerino. School of Pharmacy, Pharmacology Unit

via Madonna delle Carceri, 9 62032 Camerino (MC), Italy.

Email**:** [carlo.cifani@unicam.it](mailto:carlo.cifani@unicam.it); phone +390737403316

^#^C. Cifani, S. Gaetani equally senior authors

*A. Romano and M.V. Micioni Di Bonaventura contributed equally to this work

Supplementary Information Text

**S1 Supplementary Materials and Methods**

**S1.1 Immonostaining protocol and analyses of monoamine turnover**

After 1 day off at the end of the behavioral test of EXP.1, a first set (N = 36) of rats from the NR + S and R + S groups, received an additional 8-day cycle as reported in [1]. They were then exposed to 15 min frustration stress, and were allowed to consume highly palatable food (HPF) only for 1 h. At the end of this procedure all rats were sacrificed, their brains immediately collected, snap frozen in 2-metylbutane (-60°C) and stored at -80°C until analyzed. The brain analyses included immunohystochemical evaluation of the pattern of c-Fos expression, oxytocin receptor expression and HPLC analyses of monoamine turnover.

In particular, each brain was sliced into 20-μm serial coronal sections by using a cryostat (model HM550; Thermo Fisher Scientific, Kalamazoo, MI, USA). The coronal slices were partly mounted on positively charged slides (SuperFrost Plus, Menzel, Germany) and stored at -20°C until further analyzed for immunohystochemistry, partly microdissected into ten regions of interest that were collected in microtubes (pooled from both hemispheres), weighed to a high degree of accuracy by using a microbalance and stored at -80°C until the HPLC analysis for monoamine turnover. These regions included medial prefrontal cortex (mPFC), striatum (comprising caudate putamen (CPu), and nucleus accumbens (Acb)), hypothalamus (HYPO), amygdala (AMY), substantia nigra (SN), ventral tegmental area (VTA), dorsal raphe (DR) and locus ceruleus (LC).

**S1.1.1 Immunohystochemical analyses**

c-Fos immunohistochemistry was performed in brain areas controlling different aspects of eating and eating-related behavior that included the AMY and the paraventricular nucleus of hypothalamus (PVN), which are crucially involved in the control of stress and hedonic/homeostatic feeding respectively, different limbic areas (Acb, CPu, VTA and SN), which regulate the reinforcement and the motivational aspects of feeding, the ventral pallidum (VP) and the pedunculopontine nucleus (PP), which regulate the activity of dopamine (DA) neurons within the VTA [2]. Furthermore, a second series of sections containing the CPu and Acb was immunostained for oxytocin receptor. Briefly brain slices containing the desired brain areas underwent antigen retrieval procedure by submerging selected slides in a sodium citrate buffer (10 mM pH 6.0) heated at 95°C for 5 min [3]. Sections were then rinsed with PB (0.1 M pH 7.4) and, for c-Fos immunostaining, they were incubated for 1 h in a blocking solution containing 0.1% Triton X-100 (Sigma–Aldrich) and 2% of Normal Donkey Serum (Jackson Immunoresearch, Baltimore Pike, Pennsylvania); for oxytocin receptor, the blocking solution contained 0.05% Triton X-100 (Sigma–Aldrich) and 10% of bovine serum albumin (BSA) (SERVA Electrophoresis GmbH) and lasted 2 h. After additional washes, sections were incubated with a solution containing the primary antibodies (rabbit anti-c-Fos polyclonal primary antibody, 1:5000 dilution, Santa Cruz California, RRID: AB_2106783, for 48 h; rabbit anti-OXTR, 1:500 dilution, Alomone Labs, RRID: AB_2651123, for 24 h) at 4°C. The sections were then incubated with biotinylated donkey anti-rabbit IgG (1:500 in 0.3% PBT, Jackson Immunoresearch, Baltimore Pike, Pennsylvania, RRID: AB_2340594) for 2 h at room temperature. After incubation for 1 h with the ABC Kit (Vectastain ABC kit; Vector Laboratories), sections were stained by incubation in DAB (Vector Laboratories) chromogen solution. The slides were then rinsed with PBS, dehydrated in graded alcohol, immersed in xylene and cover-slipped with Eukitt (Sigma–Aldrich). For the semi-quantitative analysis of the slices processed by immunohystochemistry, all brain sections were observed under a Nikon Eclipse 80i microscope equipped with a colour charge-coupled device camera and controlled by the software NIS-Elements-BR (Nikon). Slices were photographed in light field using a 4× objective, and the rat brain atlas by Paxinos and Watson [4] was used as reference for the localization of the brain areas of interest. c-Fos and oxytocin receptor DAB-immunostaining were measured semi-quantitatively as optical density (OD) by using the Scion Image J software and considering, for background normalization, the averaged OD either of non-immunoreactive regions or of white matter structures within the same brain slice. For this analysis the investigator was blind to experimental groups and measurements were obtained in at least five consecutive tissue sections per animal containing the desired structure.

**S1.1.2 HPLC analysis of monoamines and monoamine metabolites extracted from brain tissues**

Brain samples obtained by microdissections from NR + S and R + S rats treated with either oleoylethanolamide (OEA) or vehicle and sacrificed 1 h after gaining access to the HPF were ultrasonicated in ice-cold 0.1 M perchloric acid and then centrifuged at 15000 × g for 20 min at 4°C as previously described [5]. Supernatants were collected and used for HPLC analyses to determine monoamine- and monoamine metabolite concentrations. We focused not only on those areas where c-Fos expression was affected by OEA treatment, but also on structures involved in the decision making, mood tone, memory processes and conditioned responses such as mPFC, HIPP (hippocampus), DR and LC, which send, respectively, serotonergic and noradrenergic inputs to the VTA [6]. Samples of selected brain regions (mPFC, Acb, CPu, HYPO, AMY, HIPP, SN, VTA, DR and LC) of all rats groups were processed simultaneously to minimize experimental errors.

DA, noradrenaline (NA), serotonin (5-HT) and both 5-HT and DA metabolites, 5-Hydroxyindoleacetic acid (5-HIAA), homovanillic acid (HVA) and 3,4-dihydroxyphenylacetic acid (DOPAC) were detected and quantified by HPLC, as previously described by [5,7]. Monoamines and their metabolites were analyzed by microbore HPLC; the detection was accomplished with a Unijet cell (BAS) with a 6 mm diameter glassy carbon electrode set at +650 mV vs an Ag/AgCl reference electrode, connected to an electrochemical amperometric detector (INTRO, Antec Leyden, Netherlands). The analytes were separated using a SphereClone 150-mm × 2 mm column (3-μm packing) and a mobile phase composed of 85 mM of sodium acetate, 0.34 mM EDTA, 15 mM sodium chloride, 0.81 mM of octanesulphonic acid sodium salt, 6% methanol (v/v) (pH = 4.85) delivered at a flow rate of 800 μl/min for a total runtime of 35 min. For each analysis, a set of standards containing various concentrations of each compound (monoamines and their metabolites) was prepared in the acid solution to obtain appropriate calibration curves. The concentrations of neurotransmitters were determined by linear interpolation from standard curves; for tissue monoamines we normalized their concentration to the weight of the wet tissue sample. Concentrations of tissue monoamines were reported in ng mg^-1^. DA and 5-HT turnover were calculated as the ratio between the metabolite and the monoamine concentrations (DOPAC+HVA/DA for the DA and 5HIAA/5-HT for the 5-HT).

**S1.2 In vivo microdialysis**

A new set of NR + S and R + S (N = 20 per group) rats were deeply anaesthetized with equithesin (3 ml kg^-1^, i.p.), placed on a stereotaxic apparatus (David Kopf Instruments) and implanted with a guide cannula (cod. MAB4.15.IC Microbiotech/se AB, Stockholm, Sweden) for microdialysis probes (cod. MAB 4.15.2.CU Microbiotech/se AB, Stockholm, Sweden) placed vertically 2 mm above the left or the right AcbSh, according to the following coordinates [4]: (AP = + 2.5 mm from bregma, ML = ± 1.2 mm from midline and DV = - 5.6 mm from skull). When the rats had fully recovered, about one week after the surgery, a probe was inserted in the guide cannula and the microdialysis was carried out in freely moving rats, according to our previous study [8]. Each microdialysis probe was perfused with a Krebs­Ringer phosphate buffer (KRP) as previously described [9] at a constant flow rate of 1.5 μl min^-1^ and dialysate samples were collected every 15 min in minivials containing 5 μl of 10% acetic acid. After an initial wash out period (at least 1 h) three baseline samples (no more than 10% difference among four consecutive samples) were collected before treating rats. Thereafter, rats were administered with OEA (10 mg kg^-1^, i.p.) or veh and after 45 min underwent the stress procedure for 15 min. At the end of this procedure they gained access to HPF for 1 h. After 30 min from the end of the HPF exposure, rats were acutely challenged with amphetamine (0.5 mg kg^-1^) administered subcutaneously. Microdialysates were collected up to 90 min after amphetamine treatment. Each microdialysis sample was analyzed by HPLC. The basal values were pooled as average of dialysate samples 1-3. The results were expressed as pg/ml, for basal values and as % of basal levels for the time-course of extracellular DA concentrations.

The correct placement of the probe was verified histologically with a post mortem Nissl staining procedure (data not shown) and data from rats with incorrect probe implant or in estrous phase were excluded from the statistical analyses.

**S1.3 In situ hybridization of oxytocin and CRF mRNA**

After 1 day off at the end of the behavioral test of EXP. 1, a second set of the same rats (N = 33) from the NR + S and R + S groups , received an additional 8-day cycle as reported in [1]. They were administered with vehicle or OEA (10 mg kg^-1^, i.p.) 45 min before the frustration stress exposure and immediately sacrificed at the end of stress. Their brains were collected, immediately snap frozen in 2-methylbutane (-60°C) and stored at -80°C until they were cut in 20 μm serial coronal sections. On these sections we performed in situ hybridization with an antisense [35S]-labelled riboprobe of rat oxytocin or with an antisense [35S]-labelled riboprobe of rat corticotropin releasing factor (CRF) that were both used in our previous studies [10–12], to detect oxytocin mRNA in the PVN and CRF mRNA in both PVN and central amygdala (CeA). The effects of OEA on oxytocin and CRF mRNA were determined by a semi-quantitative autoradiography analysis, as previously described [10–12]. For this analysis the investigator was blind to animal treatment and measurements were obtained in at least five consecutive tissues sections per animal containing the desired structure.

Briefly, oxytocin and CRF riboprobes were generated from linearized vector constructs, by in vitro transcription using SP6 RNA polymerases (Roche Diagnostic, Monza, Italy), respectively in the presence of both [^35^S]-CTP and [^35^S]-UTP. Brain sections were then hybridized at 60°C for 16 h in a buffer containing [^35^S] cRNA (~45,000 dpm ml^−1^), 10% dextran sulfate, 50% formamide, 1× Denhardt's solution, 100 μg ml^−1^ denatured salmon sperm DNA, 0.15 mg ml^−1^ tRNA and 40 mM dithiothreitol as previously described [10]. After hybridization, brain sections were exposed to Kodak Biomax film (Sigma-Aldrich) for 16 or 4 h to the detect CRF or oxytocin mRNA, respectively.

For semi-quantitative autoradiography analysis, films were first scanned (Epson perfection 3200 PHOTO) at high resolution (1200 dpi). A brain atlas [4] was used to define the localization of the brain structures of interest. Quantitative analyses of hybridized signals were performed using the Scion Image software. Optical densities were converted into radioactivity concentrations by densitometric analysis of ^14^C-microscale standards (American Radiolabeled Chemicals), so as to create for each film a calibration curve with a linear coefficient r^2^ > 0.9. In every brain section, the OD of the corpus callosum was used as background and an integrated OD value was calculated as radioactivity per extension of hybridized area. Rats in estrous phase were excluded from the statistical analyses.

**S1.4 Statistical analyses**

All data were expressed as mean ± SEM. Feeding data showed in Fig. S1B, left panel were statistically analyzed by three-way ANOVA for repeated measures, which included the intermittent food restriction (R or NR) and the frustration stress during testing (S or NS) as the between-subjects factors, with sessions time (0-15, 15-30, 30-60, 60-120 min) as the within-subject factor. Feeding data showed in Fig. S1B right panel, were statistically analyzed by two-way ANOVA with intermittent food restriction and stress as the two factors.

Feeding data showed in Fig. 1 (C-F) were statistically analyzed by one-way ANOVA with treatment as between-subject factor. Bonferroni’s test for multiple comparisons (Systat Software 10.0) was used for post hoc analyses of all feeding data.

Results obtained from immunohistochemistry, HPLC analysis of tissue monoamines and in situ hybridization were statistically analyzed by two-way ANOVA, with food restriction and treatment as the two factors. Tukey’s test was used as a post hoc to perform multiple comparisons. Moreover, for analysis of data obtained from the semi-quantitative densitometric analyses, because of the difference in the number of slices examined and the high degree of freedom, the error degrees of freedom were kept constant at a value based on the actual number of rats per group used in each experiment [3,13]. The results from microdialysis experiments resulted homoscedastic and were analyzed by two-way ANOVA for repeated measures, with time as the within variable and treatment as the between variable, followed by Dunnett’s and Bonferroni’s post hoc tests for multiple comparisons. Overall, DA extracellular levels were calculated as percentages of baseline, which was defined as the average of the first three consecutive samples with stable level of neurotransmitters. Unpaired t­test was used to evaluate the difference between the marginal means of the first three dialysate samples (basal values). In all instances, the threshold for statistical significance was set at P < 0.05.

**S2 Supplementary results**

**The combination of intermittent caloric restriction and stress exposure induced binge eating**

In agreement with our previous studies [1,14,15], rats showed body weight fluctuations during the intermittent caloric restrictions. In fact, they lost weight during each 4-day food restriction period (66% of regular chow availability) and regained it during the subsequent 4-day of *ad libitum* feeding. On the test day (day 25) of EXP. 1 the body weights of rats in the restricted and non restricted groups were not significantly different (Fig. S1A).

The statistical analyses of palatable food intakes during the test day demonstrated a significant interaction among the three factors (food restriction x stress x sessions time) (F_interaction_ = 6.902, df = 3/78, P < 0.001). Post hoc tests revealed a significant (P < 0.001) increase in HPF consumption at the 0-15 min time point in rats with a history of food restriction and exposed to frustration stress (R + S), as compared to the other groups, while no change in palatable food consumption was observed during the other sessions time of the test (15–30; 30-60; 60-120 min) among all groups (Fig. S1B, left panel). One-way ANOVA of the 120 min cumulative palatable food intake showed a two-way interaction (food restriction x stress) (F_interaction_ = 4.460, df = 1/26, P < 0.05) and the post hoc analyses (P < 0.001) revealed that R + S rats were the only group showing increased HPF intake with respect to the other groups (Figure S1B, right panel). These results demonstrated that stress exposure was able to trigger a binge-like behavior in R + S rats, which consumed a large amount of HPF within a short period of time, while it had no effect on HPF intake in rats that did not have history of food restriction.

**References**

1. Cifani C, Polidori C, Melotto S, Ciccocioppo R, Massi M. A preclinical model of binge eating elicited by yo-yo dieting and stressful exposure to food: effect of sibutramine, fluoxetine, topiramate, and midazolam. Psychopharmacology (Berl). 2009;204:113–125.

2. Floresco SB, West AR, Ash B, Moore H, Grace AA. Afferent modulation of dopamine neuron firing differentially regulates tonic and phasic dopamine transmission. Nat Neurosci. 2003;6:968–973.

3. Romano A, Gallelli CA, Koczwara JB, Braegger FE, Vitalone A, Falchi M, et al. Role of the area postrema in the hypophagic effects of oleoylethanolamide. Pharmacol Res. 2017;122:20–34.

4. Paxinos G WC. The Rat Brain in Stereotaxic Coordinates. fourth ed. San Diego: Academic Press; 1998.

5. Cassano T, Gaetani S, Morgese MG, Macheda T, Laconca L, Dipasquale P, et al. Monoaminergic changes in locus coeruleus and dorsal raphe nucleus following noradrenaline depletion. Neurochem Res. 2009;34:1417–1426.

6. Volkow ND, Wise RA, Baler R. The dopamine motive system: implications for drug and food addiction. Nat Rev Neurosci. 2017;18:741–752.

7. Bedse G, Romano A, Tempesta B, Lavecchia MA, Pace L, Bellomo A, et al. Inhibition of anandamide hydrolysis enhances noradrenergic and GABAergic transmission in the prefrontal cortex and basolateral amygdala of rats subjected to acute swim stress. J Neurosci Res. 2015;93:777–787.

8. Cassano T, Serviddio G, Gaetani S, Romano A, Dipasquale P, Cianci S, et al. Glutamatergic alterations and mitochondrial impairment in a murine model of Alzheimer disease. Neurobiol Aging. 2012;33:1121.e1-12.

9. Cassano T, Gaetani S, Macheda T, Laconca L, Romano A, Morgese MG, et al. Evaluation of the emotional phenotype and serotonergic neurotransmission of fatty acid amide hydrolase-deficient mice. Psychopharmacology (Berl). 2011;214:465–476.

10. Gaetani S, Fu J, Cassano T, Dipasquale P, Romano A, Righetti L, et al. The fat-induced satiety factor oleoylethanolamide suppresses feeding through central release of oxytocin. J Neurosci. 2010;30:8096–8101.

11. Romano A, Karimian Azari E, Tempesta B, Mansouri A, Micioni Di Bonaventura MV, Ramachandran D, et al. High dietary fat intake influences the activation of specific hindbrain and hypothalamic nuclei by the satiety factor oleoylethanolamide. Physiol Behav. 2014;136:55–62.

12. Bedse G, Romano A, Lavecchia AM, Cassano T, Gaetani S. The role of endocannabinoid signaling in the molecular mechanisms of neurodegeneration in Alzheimer’s disease. J Alzheimer’s Dis. 2014;43:1115–1136.

13. van Rijn CM, Gaetani S, Santolini I, Badura A, Gabova A, Fu J, et al. WAG/Rij rats show a reduced expression of CB₁ receptors in thalamic nuclei and respond to the CB₁ receptor agonist, R(+)WIN55,212-2, with a reduced incidence of spike-wave discharges. Epilepsia. 2010;51:1511–1521.

14. Micioni Di Bonaventura MV, Ciccocioppo R, Romano A, Bossert JM, Rice KC, Ubaldi M, et al. Role of bed nucleus of the stria terminalis corticotrophin-releasing factor receptors in frustration stress-induced binge-like palatable food consumption in female rats with a history of food restriction. J Neurosci. 2014;34:11316–11324.

15. Micioni Di Bonaventura MV, Ubaldi M, Giusepponi ME, Rice KC, Massi M, Ciccocioppo R, et al. Hypothalamic CRF1 receptor mechanisms are not sufficient to account for binge-like palatable food consumption in female rats. Int J Eat Disord. 2017;50:1194–1204.

**Figure S1**

**Fig. S1. (Panel A)** Mean ± SEM body weight (g) of female rats exposed or not exposed to repeated intermittent cycles of food restriction/refeeding. **(Panel B)** Mean ± SEM palatable food intake (kcal kg^-1^) at different sessions time (0-15, 15-30, 30-60, 60-120 min) during testing (left) and total 120 min palatable food intake (right) in the vehicle-injected rats in EXP.1. ***P < 0.001, different from the other three groups; N = 6 - 8 per group.

| **Table S1**  **Results of the two-way ANOVA analyses of c-Fos expression** | | | | |
| --- | --- | --- | --- | --- |
|  | **F _diet regimen_** | **F _treatment_** | **F _interaction_** | **df** |
| **VP** | 1.84 (P = 0.202) | 0.124 (P = 0.73) | 0.094 (P = 0.76) | 1/11 |
| **Acb** | 3.291 (P = 0.097) | 13.462 (**P < 0.01**) | 14.821 (**P < 0.01**) | 1/11 |
| **CPu** | 0.005 (P = 0.945) | 1.538 (P = 0.241) | 16.172 (**P < 0.01**) | 1/11 |
| **PVN** | 2.281 (P = 0.159) | 7.668 (**P < 0.05**) | 0.721 (P = 0.414) | 1/11 |
| **AMY** | 14.812 (**P < 0.01**) | 12.933 (**P < 0.01**) | 43.259 (**P < 0.001**) | 1/11 |
| **PP** | 0.622 (P = 0.447) | 10.574 (**P < 0.01**) | 0.039 (P = 0.847) | 1/11 |
| **SN** | 1.329 (P = 0.273) | 0.613 (P = 0.450) | 4.627 (P = 0.054) | 1/11 |
| **VTA** | 0.409 (P = 0.536) | 0.709 (P = 0.418) | 4.038 (P = 0.069) | 1/11 |

Abbreviations: Acb, nucleus accumbens; AMY, amygdala; CPu, caudate putamen; PP, pedunculopontine nucleus; PVN, paraventricular nucleus; SN, substantia nigra; VP, ventral pallidum; VTA, ventral tegmental area.

| **Table S2 Results of the two-way ANOVA analyses of brain tissue monoamines and their turnover in NR + S and R + S rats after**  **intraperitoneal injection of OEA or vehicle.** | | | | | |
| --- | --- | --- | --- | --- | --- |
|  |  | **F food restriction** | **F treatment** | **F interaction** | **df** |
| **mPFC** | **DA** | 0.009 (P=0.927) | 5.930 (**P<0.05**) | 3.705 (P=0.069) | 1/23 |
|  | **DOPAC+HVA/DA** | 1.332 (P=0.262) | 14.739 (**P<0.01**) | 7.648 (**P<0.05**) | 1/23 |
|  | **NA** | 0.045 (P=0.834) | 0.041 (P=0.842) | 0.001 (P=0.978) | 1/24 |
|  | **5HT** | 1.242 (P=0.281) | 4.512 (**P<0.05**) | 2.719 (P=0.118) | 1/20 |
|  | **5HIAA/5HT** | 0.003 (P=0.960) | 4.361 (P=0.051) | 1.818 (P=0.194) | 1/21 |
| **Acb** | **DA** | 0.449 (P=0.511) | 8.902 (**P<0.01**) | 0.023 (P=0.882) | 1/22 |
|  | **DOPAC+HVA/DA** | 0.191 (P=0.666) | 0.021 (P=0.886) | 0.890 (P=0.356) | 1/25 |
|  | **NA** | 0.000 (P=0.984) | 0.007 (P=0.933) | 0.041 (P=0.842) | 1/27 |
|  | **5HT** | 2.211 (P=0.152) | 2.175 (P=0.155) | 4.308 (P=0.050) | 1/24 |
|  | **5HIAA/5HT** | 7.405 (**P<0.05**) | 0.209 (P=0.654) | 10.034 (**P<0.01**) | 1/18 |
| **CPu** | **DA** | 0.596 (P=0.447) | 0.000 (P=0.984) | 0.107 (P=0.747) | 1/29 |
|  | **DOPAC+HVA/DA** | 0.103 (P=0.751) | 0.444 (P=0.511) | 1.600 (P=0.217) | 1/29 |
|  | **NA** | 5.602 (**P<0.05**) | 6.349 (**P<0.05**) | 0.622 (P=0.438) | 1/28 |
|  | **5HT** | 0.119 (P=0.733) | 1.279 (P=0.269) | 1.032 (P=0.320) | 1/27 |
|  | **5HIAA/5HT** | 0.000 (P=0.998) | 0.262 (P=0.613) | 0.450 (P=0.508) | 1/29 |
| **HYPO** | **DA** | 0.307 (P=0.585) | 2.715 (P=0.114) | 0.009 (P=0.926) | 1/25 |
|  | **DOPAC+HVA/DA** | 0.931 (P=0.345) | 1.570 (P=0.223) | 0.512 (P=0.482) | 1/25 |
|  | **NA** | 0.729 (P=0.402) | 11.151 (**P<0.01**) | 0.019 (P=0.891) | 1/25 |
|  | **5HT** | 17.355 (**P<0.01**) | 3.940 (P=0.062) | 0.362 (P=0.554) | 1/22 |
|  | **5HIAA/5HT** | 3.842 (P=0.065) | 2.911 (P=0.104) | 0.012 (P=0.913) | 1/22 |
| **AMY** | **DA** | 0.146 (P=0.706) | 0.468 (P=0.501) | 0.696 (P=0.413) | 1/26 |
|  | **DOPAC+HVA/DA** | 2.699 (P=0.113) | 7.952 (**P<0.01**) | 2.542 (P=0.124) | 1/27 |
|  | **NA** | 0.000 (P=0.999) | 0.826 (P=0.373) | 0.589 (P=0.451) | 1/25 |
|  | **5HT** | 2.458 (P=0.131) | 2.062 (P=0.165) | 0.270 (P=0.608) | 1/25 |
|  | **5HIAA/5HT** | 2.094 (P=0.163) | 1.285 (P=0.270) | 3.202 (P=0.088) | 1/24 |
| **HIPP** | **DA** | 0.004 (P=0.947) | 1.254 (P=0.268) | 0.071 (P=0.791) | 1/27 |
|  | **DOPAC+HVA/DA** | 0.055 (P=0.816) | 0.000 (P=0.985) | 0.688 (P=0.411) | 1/27 |
|  | **NA** | 1.317 (P=0.256) | 0.037 (P=0.847) | 0.862 (P=0.357) | 1/28 |
|  | **5HT** | 1.355 (P=0.250) | 3.698 (P=0.060) | 2.743 (P=0.104) | 1/28 |
|  | **5HIAA/5HT** | 0.395 (P=0.532) | 0.875 (P=0.354) | 0.067 (P=0.797) | 1/28 |
| **SN** | **DA** | 1.756 (P=0.198) | 0.056 (P=0.815) | 0.335 (P=0.568) | 1/27 |
|  | **DOPAC+HVA/DA** | 0.134 (P=0.717) | 2.881 (P=0.103) | 3.383 (P=0.078) | 1/27 |
|  | **NA** | 0.058 (P=0.812) | 2.591 (P=0.121) | 0.015 (P=0.905) | 1/26 |
|  | **5HT** | 0.489 (P=0.492) | 3.032 (P=0.095) | 0.040 (P=0.843) | 1/26 |
|  | **5HIAA/5HT** | 3.293 (P=0.083) | 0.668 (P=0.422) | 2.277 (P=0.146) | 1/25 |
| **VTA** | **DA** | 0.027 (P=0.871) | 12.471 (**P<0.01**) | 0.005 (P=0.942) | 1/24 |
|  | **DOPAC+HVA/DA** | 0.007 (P=0.933) | 3.643 (P=0.068) | 0.377 (P=0.545) | 1/28 |
|  | **NA** | 0.136 (P=0.716) | 5.210 (**P<0.05**) | 1.378 (P=0.251) | 1/29 |
|  | **5HT** | 0.857 (P=0.363) | 7.130 (**P<0.05**) | 2.811 (P=0.106) | 1/29 |
|  | **5HIAA/5HT** | 0.396 (P=0.535) | 0.033 (P=0.858) | 0.24 (P=0.629) | 1/29 |
| **DR** | **DA** | 0.055 (P=0.817) | 3.049 (P=0.094) | 0.278 (P=0.603) | 1/27 |
|  | **DOPAC+HVA/DA** | 2.945 (P=0.101) | 1.707 (P=0.205) | 1.428 (P=0.245) | 1/24 |
|  | **NA** | 0.031 (P=0.862) | 0.656 (P=0.427) | 0.224 (P=0.640) | 1/25 |
|  | **5HT** | 6.361 (**P<0.05**) | 2.978 (P=0.099) | 0.310 (P=0.584) | 1/23 |
|  | **5HIAA/5HT** | 0.110 (P=0.744) | 0.060 (P=0.809) | 0.353 (P=0.559) | 1/22 |
| **LC** | **DA** | 0.066 (P=0.799) | 2.962 (P=0.098) | 1.069 (P=0.311) | 1/28 |
|  | **DOPAC+HVA/DA** | 1.296 (P=0.266) | 0.196 (P=0.622) | 1.196 (P=0.285) | 1/27 |
|  | **NA** | 0.019 (P=0.892) | 1.688 (P=0.206) | 3.115 (P=0.090) | 1/27 |
|  | **5HT** | 0.575 (P=0.456) | 3.573 (P=0.071) | 0.756 (P=0.393) | 1/27 |
|  | **5HIAA/5HT** | 0.184 (P=0.672) | 0.000 (P=0.996) | 0.459 (P=0.506) | 1/24 |
| Abbreviations: Acb, nucleus accumbens; AMY, amygdala; CPu, caudate putamen; DR, dorsal raphe; HIPP, hippocampus; HYPO, hypothalamus; LC, locus coeruleus; mPFC, medial prefrontal cortex; SN, substantia nigra; VTA, ventral tegmental area. | | | | | |
|  | | | | | |
